# The Feasibility of Implementing Remote Measurement Technologies in Psychological Treatment for Depression: Mixed Methods Study on Engagement

**DOI:** 10.2196/42866

**Published:** 2023-01-24

**Authors:** Valeria de Angel, Fadekemi Adeleye, Yuezhou Zhang, Nicholas Cummins, Sara Munir, Serena Lewis, Estela Laporta Puyal, Faith Matcham, Shaoxiong Sun, Amos A Folarin, Yatharth Ranjan, Pauline Conde, Zulqarnain Rashid, Richard Dobson, Matthew Hotopf

**Affiliations:** 1 Department of Psychological Medicine Institute of Psychiatry, Psychology and Neuroscience King's College London London United Kingdom; 2 NIHR Maudsley Biomedical Research Centre South London and Maudsley NHS Foundation Trust London United Kingdom; 3 Department of Psychology King's College London London United Kingdom; 4 Department of Biostatistics & Health Informatics Institute of Psychiatry, Psychology and Neuroscience King's College London London United Kingdom; 5 Lewisham Talking Therapies South London and Maudsley NHS Foundation Trust London United Kingdom; 6 Department of Psychology University of Bath Bath United Kingdom; 7 Biomedical Signal Interpretation and Computational Simulation Group Aragón Institute of Engineering Research (I3A), IIS Aragón University of Zaragoza Zaragoza Spain; 8 Centro de Investigación Biomédica en Red of Bioengineering Biomaterials and Nanomedicine (CIBER-BBN) Madrid Spain; 9 School of Psychology University of Sussex Brighton United Kingdom; 10 Institute of Health Informatics University College London London United Kingdom; 11 Health Data Research UK London University College London London United Kingdom; 12 NIHR Biomedical Research Centre at University College London Hospitals University College London Hospitals NHS Foundation Trust London United Kingdom; 13 Institute of Psychiatry, Psychology and Neuroscience King's College London London United Kingdom

**Keywords:** depression, anxiety, digital health, wearable devices, smartphone, passive sensing, mobile health, mHealth, digital phenotyping, mobile phone

## Abstract

**Background:**

Remote measurement technologies (RMTs) such as smartphones and wearables can help improve treatment for depression by providing objective, continuous, and ecologically valid insights into mood and behavior. Engagement with RMTs is varied and highly context dependent; however, few studies have investigated their feasibility in the context of treatment.

**Objective:**

A mixed methods design was used to evaluate engagement with active and passive data collection via RMT in people with depression undergoing psychotherapy. We evaluated the effects of treatment on 2 different types of engagement: study attrition (engagement with study protocol) and patterns of missing data (engagement with digital devices), which we termed data availability. Qualitative interviews were conducted to help interpret the differences in engagement.

**Methods:**

A total of 66 people undergoing psychological therapy for depression were followed up for 7 months. Active data were gathered from weekly questionnaires and speech and cognitive tasks, and passive data were gathered from smartphone sensors and a Fitbit (Fitbit Inc) wearable device.

**Results:**

The overall retention rate was 60%. Higher-intensity treatment (*χ*^2^_1_=4.6; *P*=.03) and higher baseline anxiety (*t*_56.28_=−2.80, 2-tailed; *P*=.007) were associated with attrition, but depression severity was not (*t*_50.4_=−0.18; *P*=.86). A trend toward significance was found for the association between longer treatments and increased attrition (*U*=339.5; *P*=.05). Data availability was higher for active data than for passive data initially but declined at a sharper rate (90%-30% drop in 7 months). As for passive data, wearable data availability fell from a maximum of 80% to 45% at 7 months but showed higher overall data availability than smartphone-based data, which remained stable at the range of 20%-40% throughout. Missing data were more prevalent among GPS location data, followed by among Bluetooth data, then among accelerometry data. As for active data, speech and cognitive tasks had lower completion rates than clinical questionnaires. The participants in treatment provided less Fitbit data but more active data than those on the waiting list.

**Conclusions:**

Different data streams showed varied patterns of missing data, despite being gathered from the same device. Longer and more complex treatments and clinical characteristics such as higher baseline anxiety may reduce long-term engagement with RMTs, and different devices may show opposite patterns of missingness during treatment. This has implications for the scalability and uptake of RMTs in health care settings, the generalizability and accuracy of the data collected by these methods, feature construction, and the appropriateness of RMT use in the long term.

## Introduction

### Background

Depression is a leading cause of disability, with associated physical comorbidities and increased health care costs [1,2]. Psychological therapy is a recommended first-line treatment for mild to moderate depression [3,4]; however, approximately 50% of people do not recover following intervention [5,6]. Remote measurement technologies (RMTs) such as smartphones and wearables may assist in the treatment of depression to improve patient outcomes by detecting changes in its key behavioral aspects.

RMTs, by generating unobtrusive, continuous, and objective measures of behavior and physiology, could overcome the pitfalls of the current clinical outcome measurements, which rely on patient recall and infrequent symptom scales. Furthermore, they could help establish clinical objectives for treatment, such as a target amount of physical activity or regular sleep-time schedule and serve as indicators of whether treatments targeting particular behaviors have been effective. RMTs may also uncover digital phenotypes to identify people who are more or less responsive to certain treatments, paving the way for increased personalization of mental health care [7]. Finally, RMTs could improve patient and clinician experience of psychotherapy by strengthening communication, helping support the emotional and cognitive needs of patients and enhancing self-awareness [8].

RMTs generally apply 2 types of data collection methods: *active* and *passive*. *Active* data collection requires conscious user engagement, such as responding to mood scales, questionnaires, or speech tasks delivered to a participant’s phone. *Passive* data collection refers to the automatic capture of information via device-embedded sensors that require minimal input from users [9]; for example, accelerometers on a fitness tracker automatically detect physical activity. Used in combination, active and passive monitoring provide a way to capture continuous, ecologically valid, and high-resolution measures of signs and symptoms related to depression.

The extent to which these methods can be successfully implemented in health care and used in treatment depends on their feasibility and acceptability as tools for collecting longitudinal data in clinical populations. The feasibility of using RMTs is generally evaluated by measuring 2 broad parameters of engagement: attrition from longitudinal studies and data availability, which is the amount of usable data contributed by individuals through task completion or device use and, therefore, the opposite of missing data [10].

The measurement and reporting of attrition is relevant not only because attrition threatens the generalizability of longitudinal studies but also because it informs implementation efforts by mirroring the potential uptake and engagement within clinical settings. Much of the current research on attrition focuses on active data collection, with passive sensing being underreported. In general, studies have short follow-up periods, with systematic reviews finding a median follow-up period of 7 days for active data [11] and between 7 and 14 days for passive data [12], limiting their ability to be generalized to psychotherapy contexts, which usually span weeks. In addition, the context in which data collection occurs is key to understanding the difference in attrition rates between RMT studies. For example, a review of self-referral studies found, on average, 50% attrition in the first 15 days and varied retention rates depending on factors such as the presence and type of illness studied [13], whereas clinical trials on digital-based psychotherapy found similar attrition but at a much slower pace [14]. By contrast, large studies with dedicated recruitment resources have achieved attrition rates as low as 20% even if follow-up sessions were conducted after 2 years [15]. Therefore, if implementation of RMTs within health care is the aim, research on long-term attrition in active and passive data collection in the context of psychotherapy is critical.

Work on data availability has generally focused on active approaches [16-18], leaving passive sensing underresearched and underreported [12]. Given that both approaches require varying amounts of input and commitment from the user, *missingness* is likely to vary in the extent to which it occurs at random and may be differentially affected by individual differences [19]. This, in turn, has implications for the integrity of the constructed variables and for understanding the potential sources of biases in the data. Sparse active data points on mood questionnaires can affect how ground truth is determined, whereas less passive data can result in inaccuracies in how features are derived and the resulting data analysis (refer to Currey and Torous [20] for an example of this).

### Objective

We sought to explore the feasibility of using RMTs in a clinical setting to help uncover potential implementation and scaling issues, the resolution of which is crucial for widespread adoption. This study used a mixed methods design to evaluate the long-term engagement with active and passive approaches to the remote monitoring of mood and behavior in people with depression undergoing psychotherapy. Applying the framework developed by White et al [10], we focused on 2 forms of engagement as feasibility aspects of interest. The aims were (1) to measure engagement with the research protocol through recruitment and attrition rates, (2) measure engagement with RMTs through passive and active data availability rates and identify data streams that are more vulnerable to missing data, (3) assess the possible effect of treatment on both types of engagement, and (4) use the information gathered from qualitative interviews to aid in the explanation of the quantitative engagement data.

## Methods

### Study Design

This study was a fully remote, mixed methods, prospective cohort study with repeated measures over a 7-month period, designed to evaluate the feasibility and acceptability of using remote data collection methods in people undergoing treatment; the full protocol has been reported elsewhere [21]. The quantitative measures included recurrent clinical questionnaires and continuous digital sensor data. Qualitative measures comprised semistructured interviews that adopted an inductive approach to thematic analysis.

### Recruitment and Setting

Participants were drawn from Improving Access to Psychological Therapies (IAPT) services in South London and Maudsley National Health Service Foundation Trust, United Kingdom, a publicly funded outpatient program providing psychological treatments for adults with mild to moderate mental health disorders. IAPT services provide treatment at both high and low intensities (refer to Table S1 in Multimedia Appendix 1 for details), the allocation of which is based on several factors, including patient needs, preferences, and diagnosis. High-intensity therapy comprises approximately 10 to 12 sessions, whereas low-intensity therapy comprises approximately 6 to 8 sessions. These are usually delivered 1 week apart and can be web-based or face-to-face, depending on clinician availability and patient preference.

### Sample

A total of 66 treatment-seeking adults with depression were recruited from their local IAPT services’ waiting list, which provided the study information, and screened for eligibility either over the phone by a researcher or through a web-based self-screening tool. The sample size was determined by the primary aims and followed the general recommendation for samples of 50 to 60 participants to assess feasibility outcomes [22]. Recruitment and data collection were conducted between June 2020 and March 2022. We included adults with a current episode of depression, as measured by the Mini International Neuropsychiatric Interview [23], who owned and did not extensively share an Android (Google LLC) smartphone and were able and willing to use a wrist-worn device for the duration of the study. The exclusion criteria included a lifetime diagnosis of bipolar disorder, schizophrenia, or schizoaffective disorders, as the digital patterns of these conditions are different from those of depression, and people who were working regular night shifts or were pregnant, as these external factors can cause changes in sleep patterns. Researchers discussed health anxieties with potential participants through unstructured questions. On the basis of these discussions, those who believed that their health anxieties may worsen with continuous behavioral monitoring were excluded.

### Ethics Approval

This study was reviewed and given favorable opinion by the London Westminster Research Ethics Committee and received approval from the Health Research Authority (reference number 20/LO/0091).

### Procedures

#### Overview

**Figure 1:**
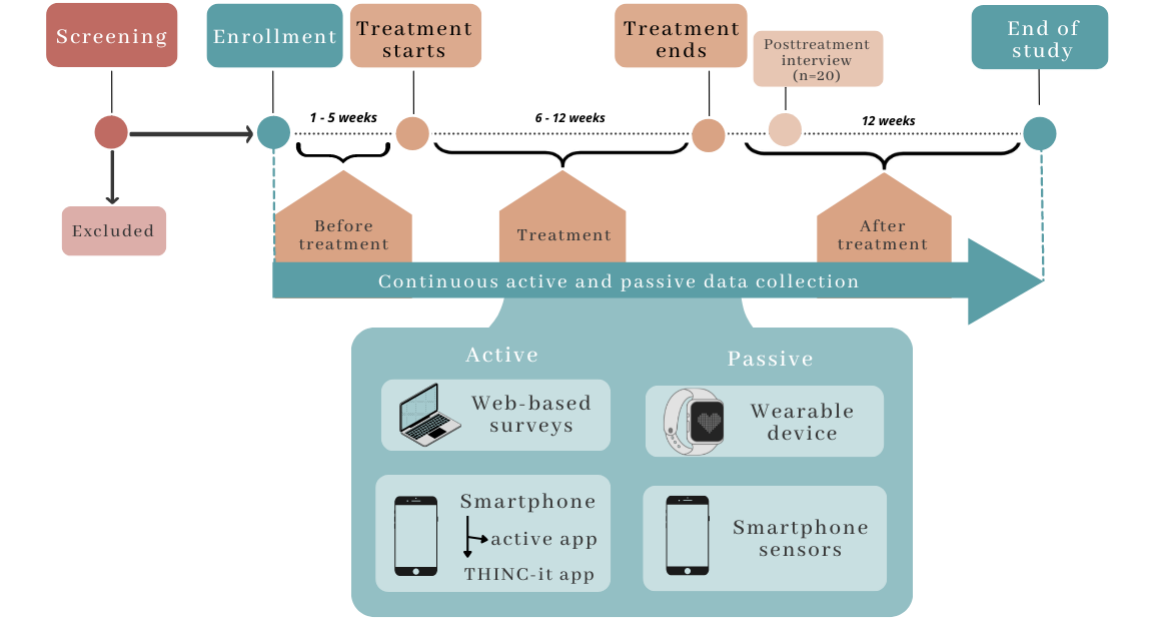
The study timeline for participants, from screening to the end of the study.

Details of the measures, technology, and procedures have been covered in depth in a previous publication [21]. The methods described in this section refer to the primary aims and outcomes of the original study protocol. Therefore, the measures presented herein are relevant to this analysis. Overall, the participants were enrolled in the study at least a week before their first therapy session. The researchers had no control over the treatment provided. Consequently, the enrolled participants had different waiting list times, treatment lengths, and treatment intensities. They were followed up throughout treatment and up to 3 months after treatment using smartphone apps and a wrist-worn device (Fitbit Charge 3 or 4 [Fitbit Inc]). Therefore, active and passive data were collected for approximately 7 months, but this depended on the treatment length, which varied from person to person. The study procedures are depicted by Figure 1.

#### Baseline Session

After providing signed informed consent, the participants provided in-depth baseline sociodemographic and clinical data related to their current and previous physical and mental health conditions, family history, treatment status, phone use, and social and physical activity levels. Clinical measures included the Patient Health Questionnaire 9-item [24], a widely validated depression questionnaire, and the Generalized Anxiety Disorder 7-item scale questionnaire [25]. Participants were then guided through the installation and setup of the 4 apps used in this study, which have been detailed in the next section.

#### Data Collection

##### Overview

Active and passive data collection began from the baseline session, and data were acquired from a variety of sources. The underlying infrastructure for data collection and storage was the Remote Assessment of Disease and Relapse (RADAR)–base platform, developed by the RADAR-Central Nervous System Consortium [26].

Passive measures were gathered from (1) Fitbit wearable device sensors and (2) smartphone sensors, and active measures were gathered from (3) web-based surveys and (4) smartphone apps.

##### Passive Measures

Passive measures were collected from 2 devices. First, the participants were provided with a Fitbit and downloaded the Fitbit app, which provided a user interface where they could track their own activity. The data extracted through the Fitbit Application Programming Interface for use in this study were related to sleep, physical activity, heart rate, and step count. The participants used their own Android smartphone and were asked to download the RADAR-base passive RMT app, a purpose-built app that collects smartphone sensor data. Only data streams with a fixed sampling rate allow for the calculation of missing data, as it provides the total number of expected data points in a period, which serves as the denominator for the total number of observed data points. These data streams were acceleration, nearby Bluetooth device detection, and GPS. GPS coordinates were obfuscated by adding a participant-specific random number as a reference point, and the relative change in location was calculated from there; therefore, an individual’s home address or precise geographic location could not be gathered.

##### Active Measures

Overall, active data were collected through 2 methods (Figure 1): web-based surveys and smartphone-based tasks sent via apps. Web-based surveys were clinical measures delivered by email via the REDCap (Research Electronic Data Capture; Vanderbilt University) software, a web-based platform for research that is conducted through a browser [27]. The smartphone-based active data were collected through (1) clinical questionnaires, (2) a series of speech tasks delivered directly to the participant’s phone via a custom-built app (the RADAR active RMT app), and (3) validated cognitive assessments in gamified format requiring a separate app, the THINC-it app [28]

Cognitive tasks were completed monthly, whereas the speech task, which required the participants to record themselves reading a short text [29] and answering a question aloud, was delivered fortnightly. All active measures were rotated weekly such that the tasks took an average of 10 minutes per week to complete, except for 1 week in a month, when the THINC-it task increased the completion time by approximately 15 minutes. The participants were notified when it was time to carry out the tasks. Details of all active measures can be found elsewhere [21].

##### Posttreatment Interview

The first 20 participants who completed the therapy and agreed to participate in an optional posttreatment interview were included in the qualitative analysis. This was a 30-minute semistructured interview conducted on the web examining the participants’ experiences of using RMTs during psychotherapy for depression. To reduce potential social desirability bias, interviews were conducted by researchers who had little to no previous contact with their interviewee.

#### Statistical Analysis

##### Quantitative Data

Quantitative data were collected regarding the following parameters of engagement:

1. Study engagement: the main outcome of attrition is defined as the division of study participants into those who completed the study (“completers”) and those who did not because of withdrawal or loss to follow-up (“non-completers”). To determine whether symptom severity at baseline was associated with attrition, 2-tailed *t* tests were performed to compare the mean severity of clinical measures taken at baseline, namely the Patient Health Questionnaire 9-item and Generalized Anxiety Disorder 7-item, across the study completion groups. The Shapiro-Wilk test was used in all cases to test for normality distributions in variables, and if this assumption was violated, nonparametric tests were used. All other assumptions for 2-tailed *t* test calculations were met.
  To test the effect of treatment characteristics on attrition, completers were compared with noncompleters in terms of treatment length and treatment intensity. The Mann-Whitney *U* test was used for the continuous variable *treatment length* given the violation of parametric assumptions, and the chi-square test was used to compare frequencies across low- and high-intensity therapy. To examine the role of overall time in the study as a confounder (given its association with treatment length and treatment intensity), we tested its potential association with attrition by conducting a 2-tailed *t* test on the mean study length across the completion groups.
2. Engagement with RMTs: engagement with RMTs was measured as the total number of data points available out of the total number of data points expected. In terms of active data, this was calculated as the number of active tasks completed out of the total number of tasks delivered. In terms of passive data, this was calculated as the number of hours in which there was at least one data point divided by the total number of hours in a day. This was then averaged to a weekly statistic.
  Logistic regression analyses were performed to assess whether being in treatment influenced the magnitude of data availability. The 3 treatment conditions were before treatment, treatment, or after treatment. Given the expected reduction in data availability over time owing to study fatigue, we adjusted these analyses for time in weeks, age, and gender. The weeks selected for analysis had to have at least 10 participants in each treatment condition; therefore, before treatment versus treatment status comparisons involved weeks 3 to 8, and after treatment versus treatment status comparisons comprised weeks 8 to 24.

##### Missing Data Thresholds

**Figure 2:**
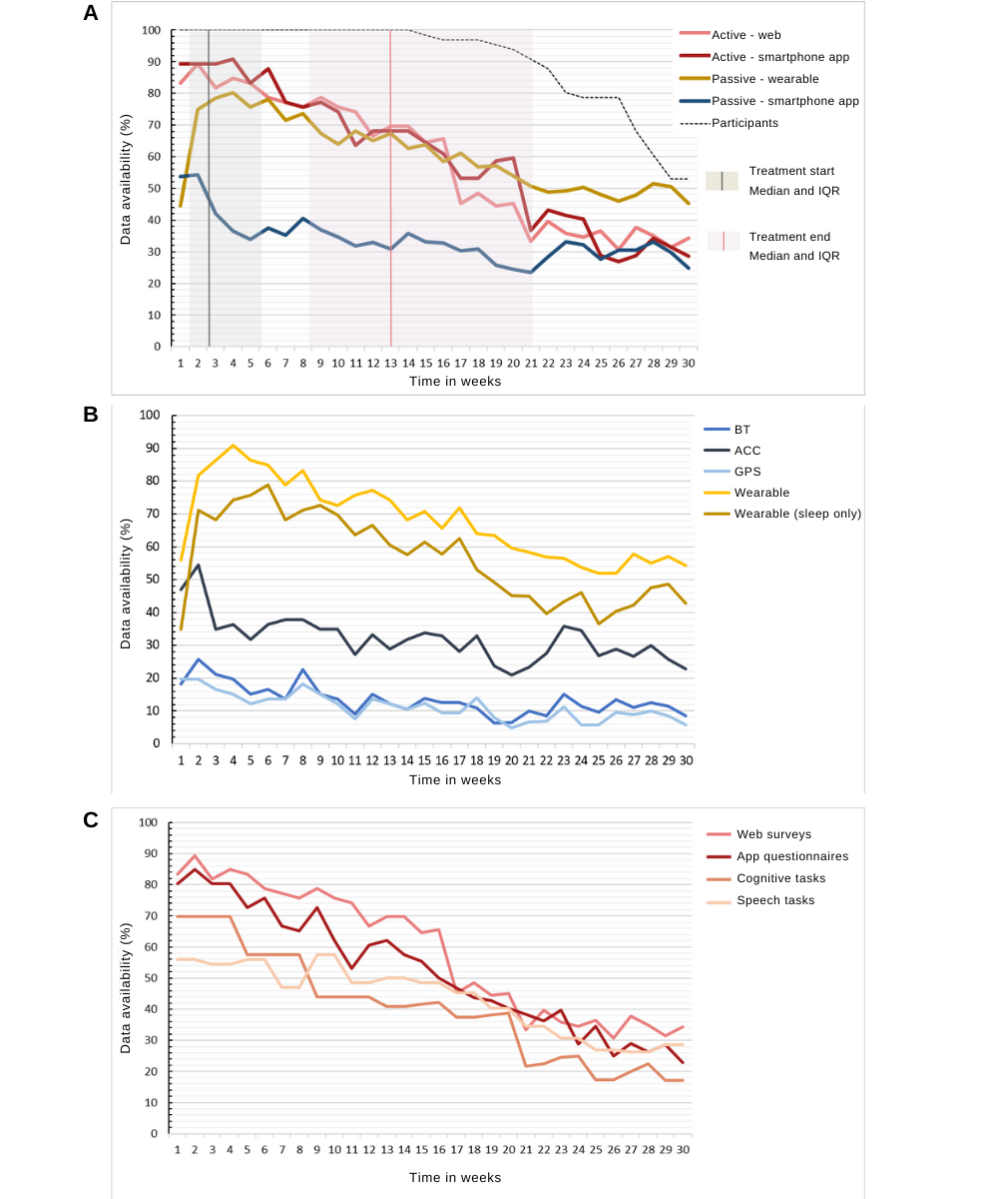
(A) Data availability by type of data. Y-axis 1 shows the percentage of people who contributed data out of the total number of available participants. Participant numbers are plotted against the secondary y-axis. (B) Data availability by passive data stream. This shows the proportion of participants with available data, averaged per week. Data were deemed available if there was at least one data point available per hour on at least 8 hours a day. (C) Data availability by active data. This shows the proportion of participants with at least one active data task completed, averaged per week. IQR: Interquartile range, ACC: accelerometer; BT: Bluetooth.

We established missing data thresholds as follows. Passive data required at least one data point per hour for at least 8 hours per day to be considered available. The total number of available hours per week were calculated, and weeks with at least 50% of available hours were deemed available. Active data were sampled weekly; therefore, active data availability for each participant was defined as the completion of at least one active task that week. The proportion of participants in the study with available data each week has been presented by the dotted line in Figure 2A.

##### Qualitative Data

Transcriptions of the recordings of the semistructured interviews were checked for accuracy by a second researcher and analyzed using a deductive approach to thematic analysis, with the iterative categorization technique [30]. The deductive approach was used in favor of an inductive approach, as certain core themes in this field have been previously reported [8,31]. These were used as initial frameworks from which to organize the initial coding, as we anticipated that these concepts would also emerge from the current data, but flexibility was given to reorganize these codes as they applied to the current data. Overarching themes, such as device engagement and the impact of treatment, were preestablished according to the quantitative objectives of the study.

All quantitative data processing and analyses were performed using R (version 4.0.2, R Core Team), and qualitative data were analyzed using NVivo (released in March 2020, QSR International).

## Results

### Study Engagement: Recruitment and Attrition

**Figure 3:**
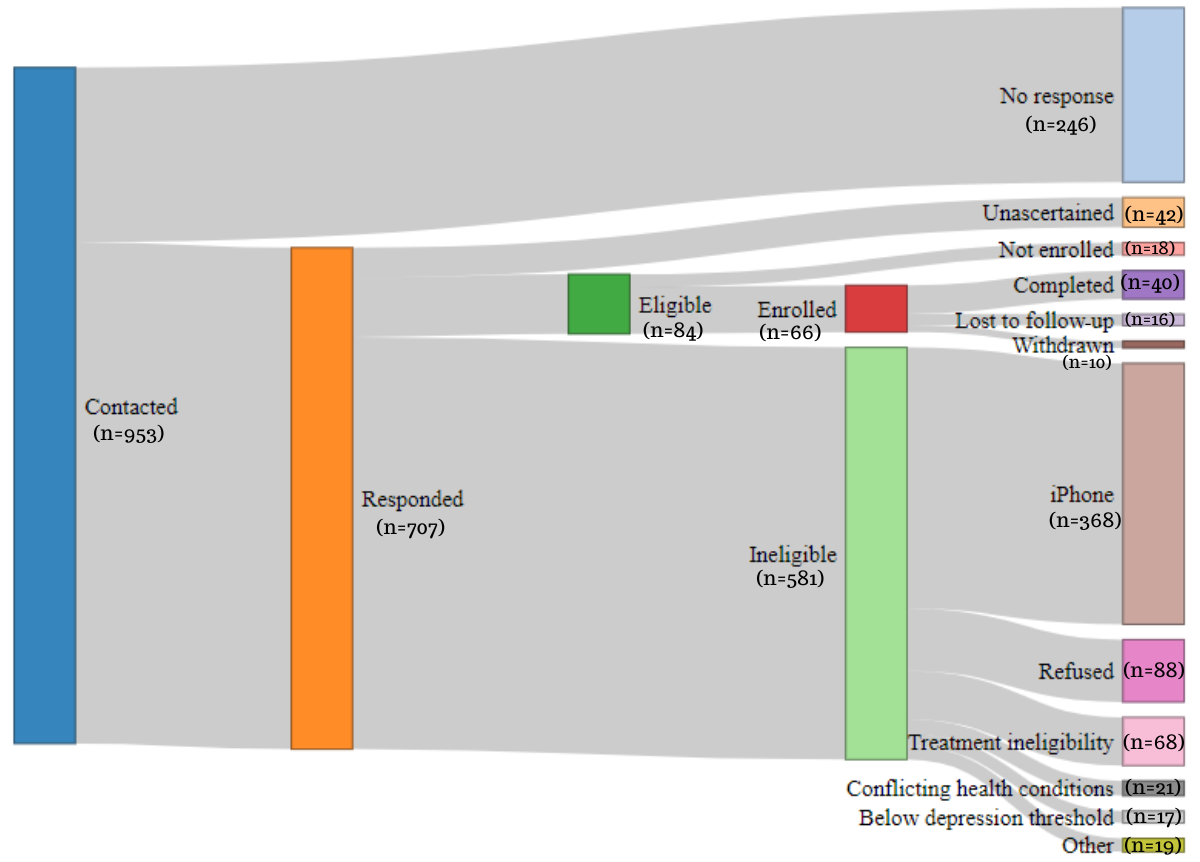
Recruitment flowchart.

Over 900 people were contacted, and of these, 66 (7.3%) were finally enrolled (Figure 3). Of the 66 enrolled individuals, 40 (61%) completed the study. Sample characteristics are presented in Table 1 and show that our sample was similar in demographic proportions to the total IAPT population in South London in terms of age, gender, ethnicity structures [32], and employment status [33].

Table 2 shows the means, medians, and proportions for those who completed the study versus those who did not on treatment-related variables. A chi-square test of independence was performed to examine the relationship between treatment intensity and attrition. The relationship between these variables was significant: N=66, *χ*^2^_1_=4.6; *P*=.03. The participants who received low-intensity treatment were more likely to complete the study than those who received high-intensity treatment.

No significant differences were found between the attrition groups across the sample characteristics of age, gender, ethnicity, educational level, employment status, and previous experience with digital health tools. A Mann-Whitney *U* test was conducted to determine whether there was a difference in treatment length between the attrition groups. The results indicated a trend toward significance in terms of the difference in treatment length between the groups (*W*=339.5; *P*=.05). A significance threshold of *P*<.05 would not regard this observation as evidence for a significant difference in treatment length between completers and noncompleters, where longer treatments would be associated with attrition. These associations cannot be accounted for by symptom severity or overall time in the study, given that study length was not associated with attrition, and the severity of depression or anxiety was associated with neither treatment length nor treatment intensity. *t* tests (2-tailed) revealed that the severity of anxiety (*t*_56_=−2.80; *P*=.007), but not depression (*t*_50_=−0.18; *P*=.86), was associated with attrition such that higher anxiety at baseline was associated with higher attrition levels (Table 2). These associations are mapped in Figure S1 in Multimedia Appendix 1.

**Table 1:** Study sample characteristics (N=66).

			Values
Age (years), mean (SD)			34.6 (11.1)
**Gender, n (%)**			
	Woman	40 (61)	
	Man	23 (35)	
	Nonbinary	3 (5)	
**Ethnicity, n (%)**			
	Asian or Asian British	2 (3)	
	Black, African, Caribbean, or Black British	11 (17)	
	Middle Eastern	1 (2)	
	Mixed or multiple ethnic groups	6 (9)	
	White British	37 (56)	
	White (other)	9 (14)	
**Education level, n (%)**			
	Secondary education	26 (39)	
	Degree-level education or diploma (eg, BSc^a^ and BA^b^)	26 (39)	
	Postgraduate degree (eg, MSc^c^, MA^d^, and PhD^e^)	14 (21)	
**Employment status, n (%)**			
	Paid employment	42 (64)	
	Unpaid employment	4 (6)	
	Unemployment	12 (18)	
	Furlough	3 (5)	
	Student	4 (6)	
	Retired	1 (2)	
Previous experience with digital health tools, n (%)			55 (83)
**Psychiatric comorbidities, n (%)**			
	0 (single psychiatric diagnosis)	17 (26)	
	1	12 (18)	
	≥2	37 (56)	
**Therapy intensity, n (%)**			
	Low intensity	33 (50)	
	High intensity	32 (48)	
**Treatment data (time in weeks), mean (SD)**			
	Treatment start lag	4.7 (4.7)	
	Treatment length^f^	11.6 (6.5)	
	Posttreatment follow-up	14.5 (6.4)	
	Total study	29.6 (6.6)	
PHQ-9^g^			16.7 (5.1)
GAD-7^h^			13.3 (4.7)

^a^BSc: Bachelor of Science.

^b^BA: Bachelor of Arts.

^c^MSc: Master of Science.

^d^MA: Master of Arts.

^e^PhD: Doctor of Philosophy.

^f^Treatment length is the number of weeks between the first and last sessions, and not the total number of sessions.

^g^PHQ-9: Patient Health Questionnaire 9-item.

^h^GAD-7: Generalized Anxiety Disorder 7-item.

**Table 2:** Summary statistics and statistical analyses of associations among completion groups, explanatory variables, and covariates.

			Attrition					Analysis						
			Completers (n=40)		Noncompleters (n=26)		Test			*df*		Test statistic		*P* value
**Treatment intensity (%)**														
	Low intensity	75.76		24.24		Chi-square test			1		4.6		.03	
	High intensity	46.88		53.13		—^a^			—		—		—	
**Treatment length (weeks), median (IQR)**														
	Length	7 (5-12.25)		12 (6-22.25)		Mann-Whitney *U* test			N/A^b^		339.5		.05	
**Covariates, mean (SD)**														
	Anxiety severity^c^	12.33 (4.66)		15.4 (4.06)		*t* test			56.28		-2.80		.007	
	Depression severity^d^	16.65 (5.08)		16.88 (5.17)		*t* test			50.40		-0.18		.86	
	Study length (weeks)	29.38 (6.02)		29.92 (7.56)		*t* test			45.02		-0.31		.76	

^a^Not available.

^b^N/A: not applicable.

^c^Anxiety was measured with the Generalized Anxiety Disorder 7-item scale questionnaire.

^d^Depression was measured using the Patient Health Questionnaire 9-item.

### Engagement With RMTs: Data Availability

The four main types of data collected were (1) wearable passive data, (2) smartphone-based passive data, (3) smartphone-based active data, and (4) web-based active data. Figure 2 shows how these data types vary in terms of their availability in the study, where 100% data completion would mean that the participants supplied, on average, 100% of the data that week. The data availability for smartphone-based passive data was between 20% and 40% for the duration of the study. Fitbit-based passive data and both active data streams had a similar proportion of data availability, but the rate of decline was lower for wearable passive data than for active data. To describe the missing data patterns across the passive data streams, the proportion of participants who provided sensor data for at least 8 hours is plotted in Figure 2B. There was no established threshold for the minimum acceptable quantity of passive data necessary to perform an analysis of these data. Therefore, we established a limit of 8 hours per day as an acceptable threshold for missing data, as the main daily activities, such as work and sleep, can be broken down into 8-hour cycles. GPS location was the passive data stream most vulnerable to missing data, followed by Bluetooth (Figure 2B). The active data stream most vulnerable to missing data for the first 10 weeks was speech and the THINC-it cognitive task thereafter (Figure 2C).

To study whether being in treatment affected data availability, the amount of data contributed by the participants who were actively receiving treatment was compared with that contributed by those who were in either pretreatment (on the waiting list) or posttreatment. Logistic regression, adjusted for time in weeks, revealed minor effects of treatment status on data availability. Significant differences in data availability were found for active smartphone and passive wearable data. When comparing those in treatment with those in pretreatment (Table 3), we found that the odds of those in treatment having active app smartphone were 2.54 times that of those on the waiting list having active app data, regardless of the time in the study. Conversely, there was a 54% decrease in the odds of contributing Fitbit data for those in treatment compared with those in pretreatment. In summary, more clinical questionnaires were completed while in treatment than while on the waiting list, but more Fitbit data were available while on the waiting list than during treatment.

**Table 3:** Odds ratios (ORs) for data availability in treatment as compared with that in pretreatment for overlapping weeks, adjusting for time (in weeks), age, and gender. A positive absolute difference in ORs show that those in treatment had more data availability.

Data streams		Treatment versus pretreatment (weeks 3-8)^a^		
		Absolute difference (unadjusted), %	OR (95% CI)	*P* value
**Active**				
	Web based	−4.40	0.89 (0.44-1.79)	.76
	Smartphone	+^b^9.24	2.34 (1.16-4.78)	.01
**Passive**				
	Wearable	−9.94	0.46 (0.21-0.95)	.04
	Smartphone	−6.31	0.72 (0.44-1.16)	.17

^a^Weeks 3 to 8 when n for all groups was <10.

^b^The absolute difference in available data (as a %) between treatment and pretreatment conditions. That is, there was 9.24% more smartphone data during treatment versus pretreatment.

### Qualitative Evaluation

**Figure 4:**
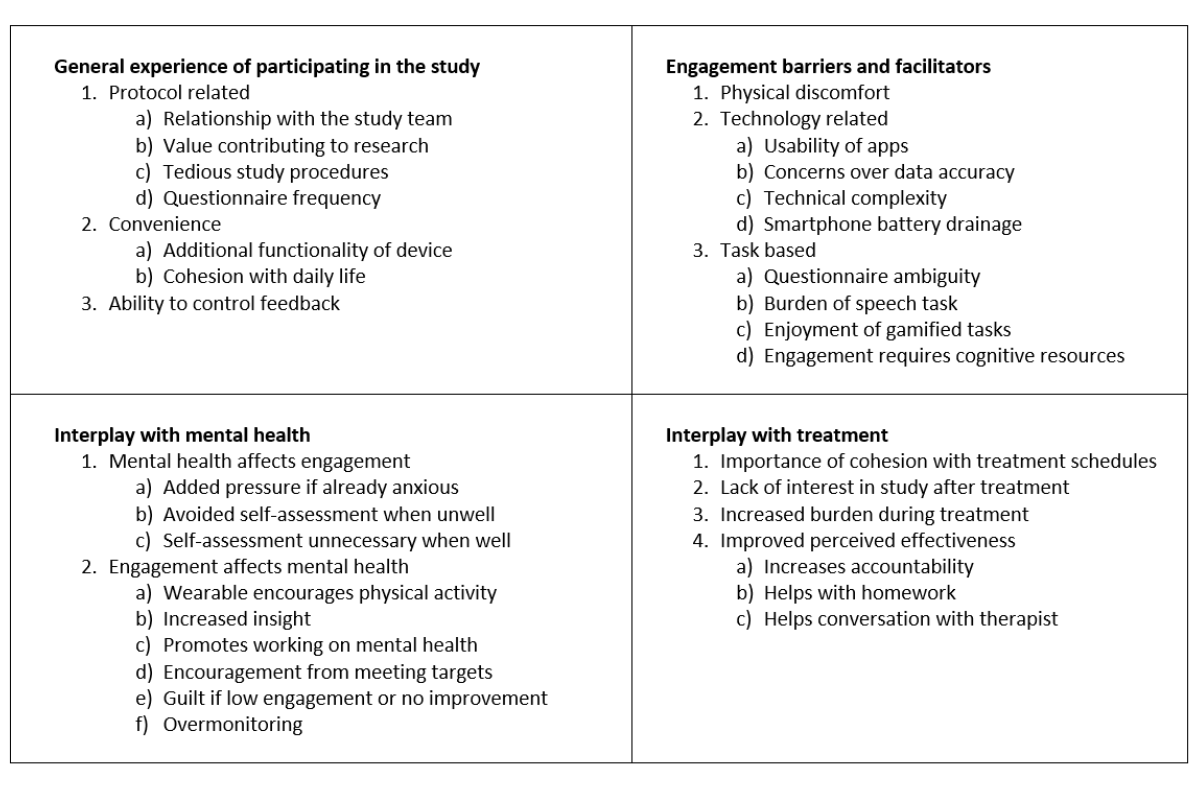
Four major themes were developed from the interviews; each subpanel shows the numbered minor themes and subthemes.

A total of 4 major themes related to the study aims were developed from the 20 semistructured interviews, as shown in Figure 4. Quotes associated with each subtheme can be found in Table S2 in Multimedia Appendix 1, and quantified participant responses are described in Figure S2.

The first major theme was the general participation experience. Protocol-related subthemes revolve around the idea that having a good relationship with the study team improves their general experience. A strong motivator of engagement was knowing that they were contributing to research, but experience was dampened by tedious study procedures in the form of repetitive and high-frequency questionnaires. Given the differences in preferences over when to receive feedback, how to receive feedback, and how much feedback to receive regarding the participants’ measured mood and behavior, it was thought that the flexibility to control these would have improved the experience.

The level of engagement with the apps and devices was affected by physical discomfort of wearing a Fitbit; technology-related issues, which relate to any technical challenges, such as battery issues and measurement accuracy; and the tasks themselves, specifically their complexity and enjoyability, which added burden or ease to their engagement.

The interplay between mental health and engagement was found to be bidirectional. For example, when participants were unwell, some reported avoiding the self-reflection required by the questionnaires, whereas others experienced this only when feeling well. On the one hand, some reported an improvement through the encouragement of health-promoting behaviors, whereas others experienced guilt or anxiety from obsessing over data, especially if no improvement was apparent.

As for the interplay between treatment and device engagement, the participants felt that the study had to integrate well with their treatment schedules. Treatment milestones were found to affect engagement with the study, with some people losing interest after therapy and others finding it harder to remain engaged with the increased burden of treatment. RMTs were seen to promote treatment effectiveness by increasing accountability with the therapist, providing targets to complete homework, and helping therapeutic conversations. The following section draws upon these results to aid the interpretation of engagement patterns.

## Discussion

### Principal Findings

We evaluated the feasibility of using both active and passive data collection methods in the psychological treatment for depression. We examined recruitment, engagement with the study protocol through attrition, and engagement with the technology through patterns of missing data. We then used qualitative interviews to help interpret the feasibility data and gain insight into such data patterns.

### Study Engagement: Recruitment and Attrition

Recruitment rates were low in proportion to the number of people initially contacted, which is in line with previous depression studies that found recruitment challenging, with large variations in success rates [34]. Digital health studies present potential participants with additional concerns, including unfamiliarity with technology and privacy misgivings [31], which may have contributed to low uptake]. Remote sensing studies tend to show a higher recruitment uptake than this study, for example, the study by Matcham et al [15], a discrepancy that could be largely explained by the exclusion of iPhone (Apple Inc) users and requirement to time recruitment with the start of treatment. Importantly, the similarity of our sample to the target population in key demographic aspects provides some reassurance that uptake was equitable across the main sample characteristics.

A retention of 60% after 7 months is markedly lower than the 94% retention after a 9-month follow-up in a sample at different stages of recurrent major depressive disorder [15], yet it is higher than the 50% retention after 15 days in a self-referral study with little researcher contact [13] and the 53% retention in studies on psychotherapy treatments [35]. Some of the key differences between these studies that can help explain the differences in engagement rates stem from the context in which the studies were carried out, participant burden, and questionnaire frequency, the latter 2 being subthemes emerging from our interviews.

We also found that treatment length and intensity affected attrition, as did higher baseline anxiety, with longer and more intense treatment being associated with early disengagement. In line with the finding from the participant interviews that participation burden increases during treatment, our quantitative analysis found that treatment characteristics and symptom severity influence retention. This could be partly because of the competing cognitive resources between engagement with RMT and treatment tasks, as described in the interviews. For example, other studies have found that constant feedback from health devices may worsen health anxieties [8], which may disproportionately impact those with higher anxiety compared with those with depression.

The main implications for engagement with the study protocol relate to scalability, generalizability, and digital divide. On the one hand, slow recruitment may reflect a low readiness among patients to sign up for remote monitoring within health care services, with implications for increased scale-up costs and staff training. Higher attrition in people with clinical and treatment-related complexities may result in them deriving fewer benefits from the implementation of RMTs than their counterparts. In addition, studies using these samples may have a higher risk of attrition bias than those on less complex treatments. This limitation to the generalizability of research findings owing to bias would, therefore, disproportionally affect those with more complex needs, widening the digital divide.

### Engagement With RMTs: Data Availability

The availability of data from the wearables and active questionnaires showed a similar pattern of decline over time. By contrast, smartphone-based passive data showed a low but stable data pattern. Some data streams, such as GPS and Bluetooth for passive data and speech and cognitive tests for active data, are more likely to be incomplete.

Some forms of data collection place a greater burden on the user than others. Therefore, it was expected that active data collection forms that have the highest participant burden would have a faster pace of decline as a function of time and cause study fatigue [36]. Therefore, it was unsurprising that the active data streams that contributed the fewest data points were speech and cognitive tasks, which were lengthier and, according to our interviews, more cognitively demanding. Although wearables require very little engagement, they still involve some level of action: they must be worn, charged, and synchronized. According to the participant interviews, 50% of those interviewed chose not to wear the Fitbit because of comfort and privacy issues, among other reasons. By contrast, passive apps are unobtrusive in their data collection, as they do not require a regular smartphone user to deviate from their usual behavior, and, therefore, produced a more stable pattern of data availability, which seemed to be less affected by study burden.

The passive data streams most vulnerable to missing data were the GPS and Bluetooth sensors. However, other passive sensing studies on mental health have found the opposite pattern, with more data being available from GPS than from accelerometers [15,20,37]. Sensor noncollection can occur for multiple reasons, including participants turning off the data permissions or the sensor itself. GPS and Bluetooth are sensors that can be easily switched off from a smartphone’s main setting page and may be seen as more intrusive forms of monitoring. This was supported by the finding that 35% of those interviewed felt “monitored” by the apps and the recurrence of “privacy” as a theme in RMT research for health care [38].

When comparing the participants in treatment with those on the waiting list, there was an increased completion of active tasks during therapy. In the current sample, the participants expressed the benefits of having a cohesive experience with RMTs and treatment such that completing active tasks during treatment helped with homework, promoted working on their mental health, and sparked conversations with their therapist. The literature on self-management in digital health shows that, despite the potential for added burden, there is a disposition for symptom tracking during treatment [39]. Conversely, people in treatment had less Fitbit wear time than those on the waiting list. The increased self-awareness that comes from tracking health with the Fitbit can be demotivating if there are no evident improvements in health outcomes such as sleep and physical activity [8], which might increase the likelihood of participants removing the device to avoid feelings of guilt and internal pressure [40].

The implications for engagement with RMTs relate to the integrity of the data collected and the differing acceptability thresholds for different devices. Low data availability means that the features derived from passive data may lack accuracy and could lead to false interpretations; however, even from the same device, different sensors contribute different amounts of data. Given that multiple sensor combinations are used to infer different aspects of behavior, accurate feature construction may require longer data collection windows for certain sensors depending on their target behavior. Although data imputation methods may help address some of these issues, increasing data availability using engagement strategies is likely to yield more accurate results. Several suggestions have been proposed by Currey and Torous [20], including overcoming the tendency of smartphones to halt data collection when apps are idle by including active components in passive apps.

Additionally, if different devices (eg, smartphones vs wearables) produce different levels of data availability, this may have implications for the appropriateness of their use, depending on the purpose and length of data collection. Smartphone-based data collection for long-term monitoring is only appropriate if the resources are available to support increased data availability strategies; otherwise, the amount of data may be too scarce to be informative. If such strategies are in place, smartphone-based data, despite having a lesser overall amount, may be a more suitable option than wearables for long-term monitoring since wearables initially provide more data, but that amount gradually decreases over time. This study demonstrated wearables to be a feasible method for collecting activity and sleep data, 2 core items in psychotherapy for depression, for at least 32 consecutive weeks, before data availability falls below the 40% mark. Therefore, this method may be superior to smartphone-based data in a naturalistic context involving relatively longer-term treatments. However, it is important to consider strategies to increase user engagement with technology that take a patient-centered approach, including selecting measures that are meaningful to patients [41].

### Limitations and Future Directions

This was a longitudinal cohort study, so the comparison groups of treatment versus nontreatment differed in more ways than only the exposure to treatment and different treatment intensities. The participants in the treatment group were compared with those in the same week of the study but who had yet to start treatment; delayed treatment start was related to treatment intensity, clinical risk, catchment area for the health care center, and symptom severity. Despite our efforts to account for these variables in the analysis, there is a possibility of residual confounders. Future studies could quantify the components of treatment that are related to poorer engagement.

Engagement with RMTs is broadly defined as data availability, which assumes that the occurrence of missing data is because of the participants deliberately disengaging. However, despite presenting some evidence of personal and clinical characteristics related to data availability, missing data can also be completely missing at random because of software errors. These factors may affect data streams differently based on technical factors. Future research could determine the nature of missingness by mapping technical issues to missing data.

There is no standard method to establish a threshold for “missing data.” In this study, we justify a minimum of 8 hours of passive data and at least one active task completed; however, other studies (eg, the study by Matcham et al [15]) considered data availability as a single point of data per hour. It is critical to understand how much missing data are admissible before the integrity of the data is affected so that there can be an accurate characterization of the behavior. A single point of active data may describe a symptom experience for the previous 2 weeks, whereas a single passive data point covers a second’s worth of activity. Therefore, rates of missingness need to be interpreted with this relativity in mind, and future studies should work toward establishing acceptable thresholds for data availability for each behavioral feature under study.

This study has shown engagement differences between data collection types and a difference in engagement between those in treatment and those on the waiting list; however, despite the statistical significance, future work should attempt to establish whether these differences are clinically meaningful.

Finally, the COVID-19 pandemic has given rise to a rapid adoption of technology, especially in the health care sector [42]. This is likely to have had an impact on people’s attitudes toward digital tools for health monitoring and, consequently, engagement with RMTs. As a result, this study may have picked up on higher technology acceptance, or conversely, technology fatigue, as a factor of time.

### Conclusions

We investigated the feasibility of remote collection methods in the psychological treatment for depression and reported the extent to which it was feasible to collect active and passive data via RMTs in a population with depression within a health care setting. Uptake was low but equal across the main demographic categories and, therefore, broadly representative of the target population. Retention in our study was low, but comparable with retention rates in psychological therapy [35]. Treatment characteristics such as length and intensity were associated with attrition, as was higher baseline anxiety, suggesting that patients undergoing more complex treatment may perceive fewer benefits from long-term remote monitoring. In addition, different data streams showed different levels of missing data despite being gathered from the same device, implying that different sensors may require different data collection protocols to ensure sufficient data for accurate feature construction. Being in treatment also affected RMT engagement in different ways, depending on the device, with Fitbit contributing less data during treatment but active tasks being completed more often. Future work should establish acceptable thresholds for data availability for different sensors and devices to ensure a minimum requirement for the integrity of RMT data and investigate which aspects of treatment are related to poorer engagement. Finally, successful implementation of RMTs requires more than the user engagement measures presented in this study; however, adopting these user engagement measures is a key first step.

## Appendix

Multimedia Appendix 1 Supplementary information to the paper, including information on treatments and quantitative and qualitative analyses.

## Abbreviations

IAPT: Improving Access to Psychological Therapies
RADAR: Remote Assessment of Disease and Relapse
REDCap: Research Electronic Data Capture
RMT: remote measurement technology
