# Supplementary material for: The Feasibility of Implementing Remote Measurement Technologies in Psychological Treatment for Depression: Mixed Methods Study on Engagement
Source: JMIR Ment Health. 2023 Jan 24;10:e42866. doi: 10.2196/42866 (PMC9906314; doi:10.2196/42866)
Supplement: Multimedia Appendix 1 [file mental_v10i1e42866_app1.docx]

# SUPPLEMENTARY INFORMATION

## **Supplementary Table 1 -** Psychological interventions delivered by Improving Access to Psychological Therapies (IAPT) for depression and anxiety disorders. Taken from the National Collaborating Centre for Mental Health (2018).

|  | Condition | Psychological therapies |
| --- | --- | --- |
| Step 2:  Low intensity interventions | Depression | Individual guided self-help based on CBT, Computerised CBT,  Behavioural Activation, Structured group physical activity programme. |
|  | Generalised anxiety disorder | Self-help, or Guided self-help, based on CBT, Psycho-  educational groups, Computerised CBT |
|  | Panic disorder | Self-help, or Guided self-help, based on CBT, Psycho-  educational groups, Computerised CBT |
|  | Obsessive compulsive disorder | Guided self-help based on CBT |
| Step 3:  High intensity interventions | Depression    For individuals with mild to moderate severity who have not responded to initial low-intensity interventions | CBT (individual or group) or IPT    Behavioural Activation    Couple therapy^a^    Counselling for depression    Brief psychodynamic therapy    **Note:** Psychological interventions can be provided in combination with antidepressant medication. |
|  | Depression  Moderate to severe | CBT (individual) or IPT, each with medication |
|  | Depression  Prevention of relapse | CBT or mindfulness-based cognitive therapy^b^ |
|  | Generalised anxiety disorder | CBT, Applied relaxation |
|  | Panic disorder | CBT |
|  | PTSD | Trauma-focused CBT, Eye  Movement Desensitisation and Reprocessing^c^ |
|  | Social anxiety disorder | CBT specific for social anxiety disorder^d^ |
|  | OCD | CBT (including exposure and response prevention) |

^a^ If the relationship is considered to be contributing to the maintenance of the depression, and both parties wish to work together in therapy. IAPT recognises two forms of couple therapy and supports training courses in each. One closely follows the behavioural couple therapy model. The other is a broader approach with a systemic focus.

^b^ CBT during treatment in the acute episode and/or the addition of mindfulness-based cognitive therapy when the episode is largely resolved. Mindfulness is not recommended as a primary treatment for an acute depressive episode.

^c^ If no improvement, an alternative form of trauma-focused psychological treatment or augmentation of trauma-focused psychological treatment with a course of pharmacological treatment.

^d^ Based on the Clark and Wells model or the Heimberg model.


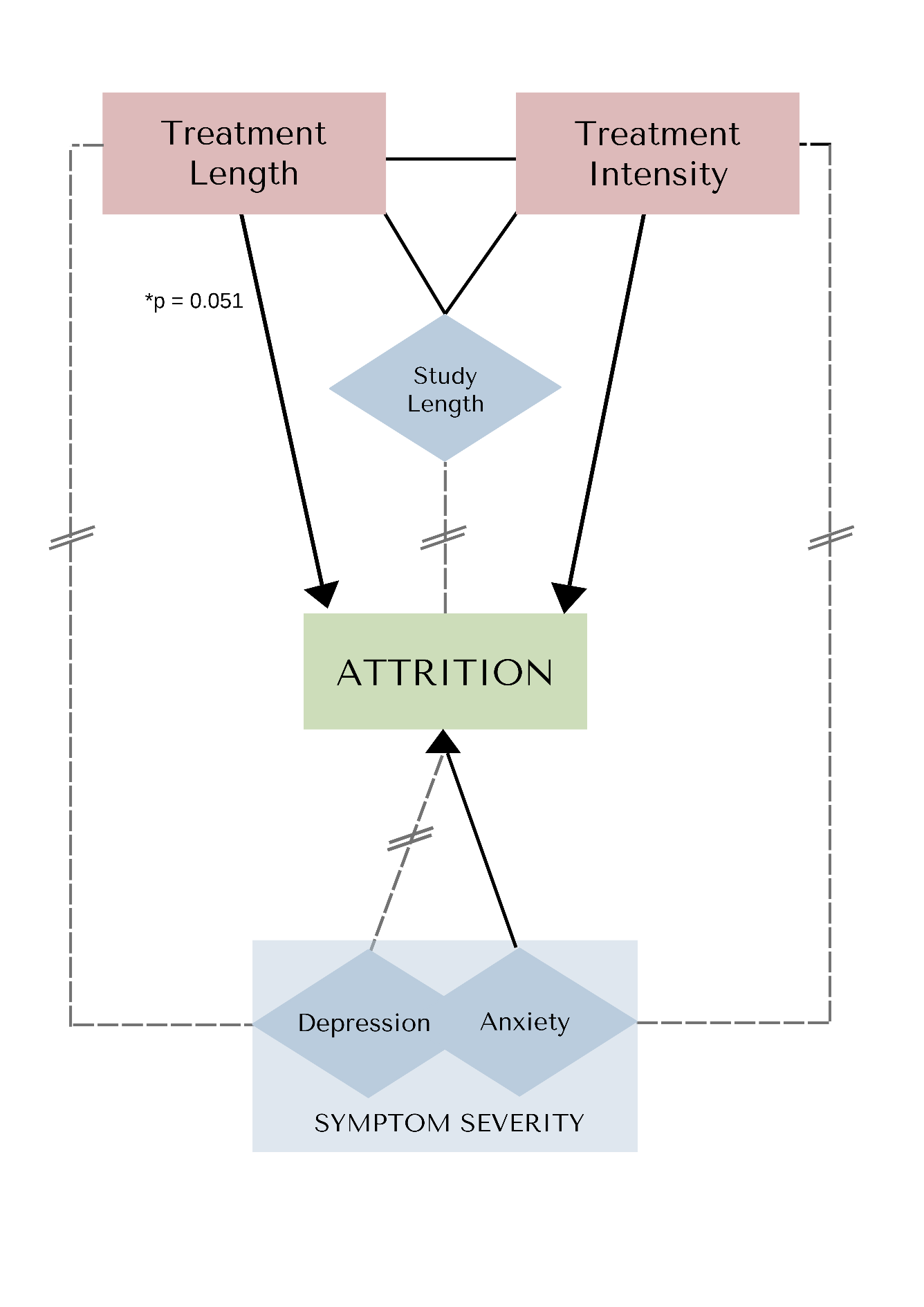


**Supplementary Figure 1 | Associations between the main outcome of attrition, exposure variables and covariates.** Solid lines represent statistically significant (p< 0.05) associations while dotted lines represent no significant associations between the variables.

## **Supplementary Table 2 -** Four major themes were developed from the interviews; each panel shows the numbered minor themes and subtheme.

| **General Experience of Participating**   1. Protocol-related    1. Relationship with study team    2. value contributing to research    3. tedious study procedures    4. questionnaire frequency 2. Convenience    1. additional functionality of device    2. cohesion with daily life 3. Ability to control feedback | **General Experience of Participating**   1. Protocol-related 2. *“When you have the access, I’ve had to communicating with [the study team], it hasn't been difficult […] And yeah, uh, just feeling that this is a supportive team as well, and that um you're speaking with people and that there is compassion there, has been really helpful”* 3. *“Basically, I am happy to do anything and everything that's helpful for um the study, for mental health patients and practitioners, and doctors and therapists and anyone who is in it to help you know.”* 4. *“I mean in general I think questionnaires are tedious as I said, long questionnaires. I was happy on one hand to have them weekly, but on the other hand um I found them long.”*   *“It felt a bit too similar, um, every day. You know, ‘how did you sleep?’, ‘Yes, OK’, you know? It's like the same sort of questions every day so it's a little bit monotonous I think.”*   1. *“Sometimes I- I had a bit of a back log after for a couple of days when I just haven't had a chance [to do every questionnaire].”* 2. Convenience    1. *“…just to track stuff such as, uh, like my eating, like how much water that I intake and like, my sleep patterns.*    2. *“Sometimes I sort of had days where I just haven't had time to do the questionnaires, or I've sort of got distracted with something else.”* 3. Ability to control feedback   *“Um, maybe not too much information because sometimes too much can be a bit of an overload, but some kind of information, like over the course of like a fortnight or like a month or something like that.”*  *“Yeah, I think the problem with choosing what to send and stuff is like, I, you don't always know what's relevant.”* |
| --- | --- |
| **Engagement barriers and facilitators**   1. Physical discomfort 2. Technology-related    1. usability of apps    2. concerns over data accuracy    3. technical issues    4. battery drainage 3. Task-based    1. questionnaire ambiguity    2. burden of speech task    3. enjoyment of gamified tasks    4. engagement requires cognitive resources | **Engagement barriers and facilitators**   1. Physical discomfort   *“The strap that came with it, it's sort of- it really- I got like um, bruises and marks on my hands, my arm from - erm, I don't know if I was allergic to it or- or what I don't know, but it was, it was quite painful.”*  *“They're quite bulky so they're not very comfortable to wear at night”.*   1. Technology-related 2. *“The apps were, like, very accessible, were very easy to use.”* 3. *“I hope me on purpose not wearing [the device] sometimes, didn't like to distort the data.”* 4. *the glitchy app at the beginning, so you know like when I was- I was opening like, literally when I was doing anything on my phone it would just keep popping open, just keep popping open and it was quite annoying* 5. *“Passive app was a huge power hog. Erm, I don't know if there's a memory leak in it or something, but I often found I actually had to kill it to get- to stop my phone haemorrhaging battery.”* 6. Task-based 7. *“I can't remember how they are phrased, but sometimes, I didn't know if I was answering correctly.”* 8. *“The most burdensome, I think it’s doing the speaking test just because you need to be in a quiet space. Uh, and it sort of feels like the barrier to doing that is a bit higher. Obviously when I do it, you know, it just takes me a couple of minutes and that's fine. I can just step out of the room and do it. But the mental barriers to doing that feel bigger.”* 9. *“The cognitive games that you have to play. You know they- they were really interesting, just ended up playing them for fun.”* 10. *“Because when I was doing [the active tasks] I was really struggling to focus and it was like, it was like a real challenge just to sort of sit still to do it and to really focus."* |
| **Interplay with Mental Health**   1. Mental health affects engagement    1. added pressure if already anxious    2. avoided self-assessment when unwell    3. self-assessment unnecessary when well 2. Engagement affects mental health    1. Wearable encourages physical activity    2. increased insight    3. promotes working on mental health    4. encouragement from meeting targets    5. Guilt if low engagement or no improvement    6. Over-monitoring | **Interplay with Mental Health**   1. Mental health affects engagement 2. *when I was very anxious, it would just be another thing to do. You know, it was just part of my list and my ever-growing lists, you know, that I felt like I was running after each task.* 3. *“When I did have very very low days. I didn't want to open the questionnaires; I didn't want to have to assess myself while I'm feeling bad.”* 4. *“Some weeks I actually found it made it harder because I was in such a good mood, I was more likely to go out and enjoy myself and I'll forget about my tasks […] or I was in too good of a mood to focus on doing anything a bit more serious.”* 5. Engagement affects mental health 6. *‘Did it affect my behaviour at all just having it?’, I would say yes. As I said, like with the, you know, being able to like, see my heart rate and stuff and just even encouraging me to maybe do some more exercise.”* 7. *“I think if I wasn't wearing the Fitbit, I’d definitely be a lot less aware of my daily habits. Um, like all of the things that kind of result in a person being like good of health, physically and mentally, I'd be less aware of those things.”* 8. *“I just think because being in the study has kind of felt like you are sort of proactively working on your own mental health.”* 9. *“Obviously, it's also good that when I'm out and about it sort of counts how many steps I do, so I've got that target to sort of work towards.”* 10. *“You know, I do feel a bit guilty, the fact that I haven't really paid much attention to it in the last sort of month or two, which is, you know, it's terrible”*   *“I didn't feel very well about evaluating my sleep, especially because I have insomnia. So just saying every day that I haven't had enough sleep and that it wasn't good quality was not really a great start to my day.”*   1. *“I think I probably would have got a bit too caught up in that if I was being given updates of where I was at with my stats.”*   *“Perhaps a Fitbit could be almost quite negative, but, because I think you could maybe get a little bit obsessed with it.”* |
| **Interplay with treatment**   1. Importance of cohesion with treatment schedules 2. Lack of interest in study after treatment 3. Increased burden during treatment 4. Improved perceived effectiveness    1. increases accountability    2. helps with homework    3. helped conversation with therapist | **Interplay with treatment**   1. Importance of cohesion with treatment schedules   *“When you start to get worse, like it’s just disheartening. And then you have to go two - twice through it, one for the therapist and one for the study. So that wasn’t great.”*   1. Lack of interest in study after treatment   *“I think after treatment, I found it a lot more difficult [to participate], I think, also because I was kind of moving on from therapy and I don't know if maybe that kind of included like, I need to go back and you know, keep looking at my mental health for the survey etc. But for some reason I did find the post therapy the hardest part to continue with this study, I think.”*   1. Increased burden during treatment   *“I would say it was probably slightly more difficult to participate when I was having the treatment because although I felt- although motivating me to do it, I guess kind of like you have homework from the CBT and then you sort of have extra things to do.”*   1. Improved perceived effectiveness    1. *“You could have it where like the therapist can see the log and they could see like ‘Oh, you did some activity at you know 5:30 when you finish work so I can see that you went for walk this day or maybe you didn't go this day.’. It stops people like me lying. (laughs)”*    2. *“My homework once was, because of the lockdown there, they suggested, like I, uh, go for walks after work so […] for example, you could hypothetically implement that (inaudible) Fitbit into that so [therapists] can actually see.”*    3. *“It felt like it's just part of that - a process of evaluation, and the data was helpful when I needed to speak to my therapist about things.”* |

**Supplementary Figure 2 | Quantified interview responses.** *Bar chart showing the proportion of people providing positive, negative, or neutral responses to 12 interview items.*

**References**:

National Collaborating Centre for Mental Health. (2018). The improving access to psychological therapies manual. *UK: NCCMH*. <https://www.england.nhs.uk/wp-content/uploads/2018/06/the-iapt-manual-v5.pdf>
